# Large-scale phosphoproteome analysis in wheat seedling leaves provides evidence for extensive phosphorylation of regulatory proteins during CWMV infection

**DOI:** 10.1186/s12870-023-04559-3

**Published:** 2023-11-02

**Authors:** Lu Chen, Jin Yang, Haichao Hu, Yaoyao Jiang, Lixiao Feng, Jiaqian Liu, Kaili Zhong, Peng Liu, Youzhi Ma, Ming Chen, Jian Yang

**Affiliations:** 1 Institute of Crop Sciences, State Key Laboratory of Crop Gene Resources and Breeding, National Key Facility for Crop Gene Resources and Genetic Improvement, Chinese Academy of Agricultural Sciences (CAAS), Beijing, 100081 China; 2 State Key Laboratory of Plant Physiology and Biochemistry, College of Biological Sciences, China Agricultural University, Beijing, 100193 China; 3 State Key Laboratory for Quality and Safety of Agro-Products, Institute of Plant Virology, Ningbo University, Ningbo, 315211 China

**Keywords:** *Chinese wheat mosaic virus* (CWMV), Protein phosphorylation, Wheat, Plant immunity, Plant-pathogen interaction

## Abstract

**Background:**

*Chinese wheat mosaic virus* (CWMV) often causes severe damage to wheat (*Triticum aestivum* L.) growth and yield. It is well known that a successful infection in plants depends on a complex interaction between the host plant and the pathogen. Post-translational modification (PTM) of proteins is considered to be one of the main processes that decides the outcome of the plant-pathogen arms race during this interaction. Although numerous studies have investigated PTM in various organisms, there has been no large-scale phosphoproteomic analysis of virus-infected wheat plants. We therefore aimed to investigate the CWMV infection-induced phosphoproteomics changes in wheat by high-resolution liquid chromatography-tandem mass spectroscopy (LC–MS/MS) using affinity-enriched peptides followed by comprehensive bioinformatics analysis.

**Results:**

Through this study, a total of 4095 phosphorylation sites have been identified in 1968 proteins, and 11.6% of the phosphorylated proteins exhibited significant changes (PSPCs) in their phosphorylation levels upon CWMV infection. The result of Kyoto Encyclopedia of Genes and Genomes (KEGG) enrichment analysis showed that most of the PSPCs were associated with photosynthesis, plant-pathogen interactions, and MAPK signaling pathways. The protein–protein interaction (PPI) network analysis result showed that these PSPCs were mainly participated in the regulation of biosynthesis and metabolism, protein kinase activities, and transcription factors. Furthermore, the phosphorylation levels of TaChi1 and TaP5CS, two plant immunity-related enzymes, were significantly changed upon CWMV infection, resulting in a significant decrease in CWMV accumulation in the infected plants.

**Conclusions:**

Our results indicate that phosphorylation modification of protein plays a critical role in wheat resistance to CWMV infection. Upon CWMV infection, wheat plants will regulate the levels of extra- and intra-cellular signals and modifications of enzyme activities via protein phosphorylation. This novel information about the strategies used by wheat to resist CWMV infection will help researchers to breed new CWMV-resistant cultivars and to better understand the arms race between wheat and CWMV.

**Supplementary Information:**

The online version contains supplementary material available at 10.1186/s12870-023-04559-3.

## Background

Proteotype is the proteomic state of a cell and represents the cell’s genotype, developmental history, and growth environment [1]. Protein PTM can function at various steps, including cotranslational modification, to enablers of location, function, and signaling, and finally to markers for stability and degradation [2]. To date, PTMs have been shown to add small proteins or domains to specific proteins to allow them to have additional function(s) in ubiquitination, lipidation, glycosylation, methylation, phosphorylation, or acetylation [3–5]. PTM also ensures that the modified proteins have more precise functions to regulate the expressions and functions of host genes. Protein phosphorylation is one of the most well-known PTMs in cells, representing approximately 53.5% of all reported PTMs [6].

Crop plants are constantly attacked by pathogenic bacteria, fungi, and viruses [7]. Many recent reports have demonstrated that phosphorylation of signal transduction-associated proteins can enhance plant defense against pathogen invasions [8]. Many proteins are known to contain multiple phosphorylation sites, and phosphorylation of specific sites can amplify the functions of the phosphorylated proteins [9]. Many disease resistance-associated proteins can be phosphorylated during pathogen invasions. For example, BOTRYTIS-INDUCED KINASE 1 (BIK1) is a receptor-like cytoplasmic kinase (RLCK) and undergoes hyper-phosphorylation upon PAMP perception to function as a signaling hub immediately downstream of PRRs [10]. To date, many BIK1 substrates have been identified. BIK1 can phosphorylate RESPIRATORY BURST OXIDASE HOMOLOGUE D (RBOHD), Ca^2+^-permeable channel OSCA1.3, and CYCLIC NUCLEOTIDE-GATED CHANNEL (CNGC) proteins to fine-tune host immune signaling [11–15]. Therefore, protein phosphorylation has been considered the most common and intensive PTM involved in host defense signaling. Because of the importance of protein phosphorylation in plant defense signaling, numerous studies have been done in the past few decades, including identifications of phosphorylated proteins, map phosphorylation sites in target proteins, quantify phosphorylation levels, and functional characterizations of phosphorylated proteins. More recently, phosphoproteomics has become a popular approach to analyzing various biological regulatory networks involved in protein phosphorylation at the global level [16]. In addition, improved Liquid Chromatography-Mass Spectrometry (LC/MS)-based techniques have allowed researchers to determine the roles of phosphorylated proteins in plant growth and stress responses. For example, Zeng and others found extensive phosphorylation of proteins associated with bast fiber growth in ramie using a phosphoproteomic analysis approach [17]. Ma and others also conducted proteomic and phosphoproteomic analyses and revealed a complex signaling network that is responsive to pollen abortion [18]. To better understand the global protein phosphorylation in the effector-triggered immunity (ETI), Yasuhiro and colleagues did a phosphoproteomic screen using *Arabidopsis thaliana* as the model plant and identified 109 differentially phosphorylated residues in the membrane-associated proteins [19]. In a separate report, Yu and others reported 255 phosphorylated proteins and 79 phosphopeptides involved in Ca^2+^ signaling, reactive oxygen species (ROS), mitogen-activated protein kinase (MAPK) cascade, and hormone signaling-associated phosphopeptides, respectively, during both PTI and ETI [20].

Wheat (*Triticum aestivum* L.) is a major food crop in many countries, and its quality and yield are often affected by various abiotic and biotic stresses [21]. Several soil-borne viruses can infect wheat plants in the field and affect wheat growth and yield [22]. CWMV is an important wheat disease in China and is mainly transmitted by the soil-borne fungus *Polymyxa graminis* [23]. The young leaves of the CWMV-infected wheat plants often show light chlorotic streaking, while the mature leaves may have strong chlorotic streaking [24]. CWMV is a member of the genus *Furovirus*, family *Virgaviridae*, and has a genome consisting of two positive sense single-stranded RNAs (RNA1 and RNA2) [25, 26]. CWMV RNA1 consists of 7147 nucleotides (nt) and has three major predicted open reading frames that encode three proteins that are important to viral replication and transmission. CWMV RNA2 is 3564nt long and encodes four proteins: the 19 kDa major coat protein (CP), two CP-related proteins (23 kDa N-CP and 84 kDa CP-RT), and a 19 kDa cysteine-rich protein (CRP, known as an RNA silencing suppressor) [27–29]. The S162 and S165 in the CRP can be phosphorylated by SAPK7 and the phosphorylated CRP plays a key role in CWMV infection. Phosphorylation of CRP can also inhibit H_2_O_2_ production and cell death through alteration of the TaUBA2C chromatin-bound status and attenuation of its RNA and DNA binding activities [30].

Based on the current knowledge, we hypothesized that phosphorylation of cellular proteins may also significantly affect CWMV infection and pathogenesis in infected host cells. So far, only a few studies have been made to elucidate the arms race between CWMV and its hosts, and only a few post-translationally modified host proteins have been identified in the CWMV-infected host plants. In this study, we performed a phosphoproteomic analysis to identify the phosphorylated peptides in wheat seedlings during CWMV infection through a combination of 2D-LC–MS/MS and bioinformatics analysis. The findings presented in this paper will provide new knowledge on the mechanism controlling CWMV infection in wheat, and possibly in other monocots.

## Results

### Large-scale phosphoproteomic analysis using CWMV-infected wheat seedlings

**Fig. 1:**
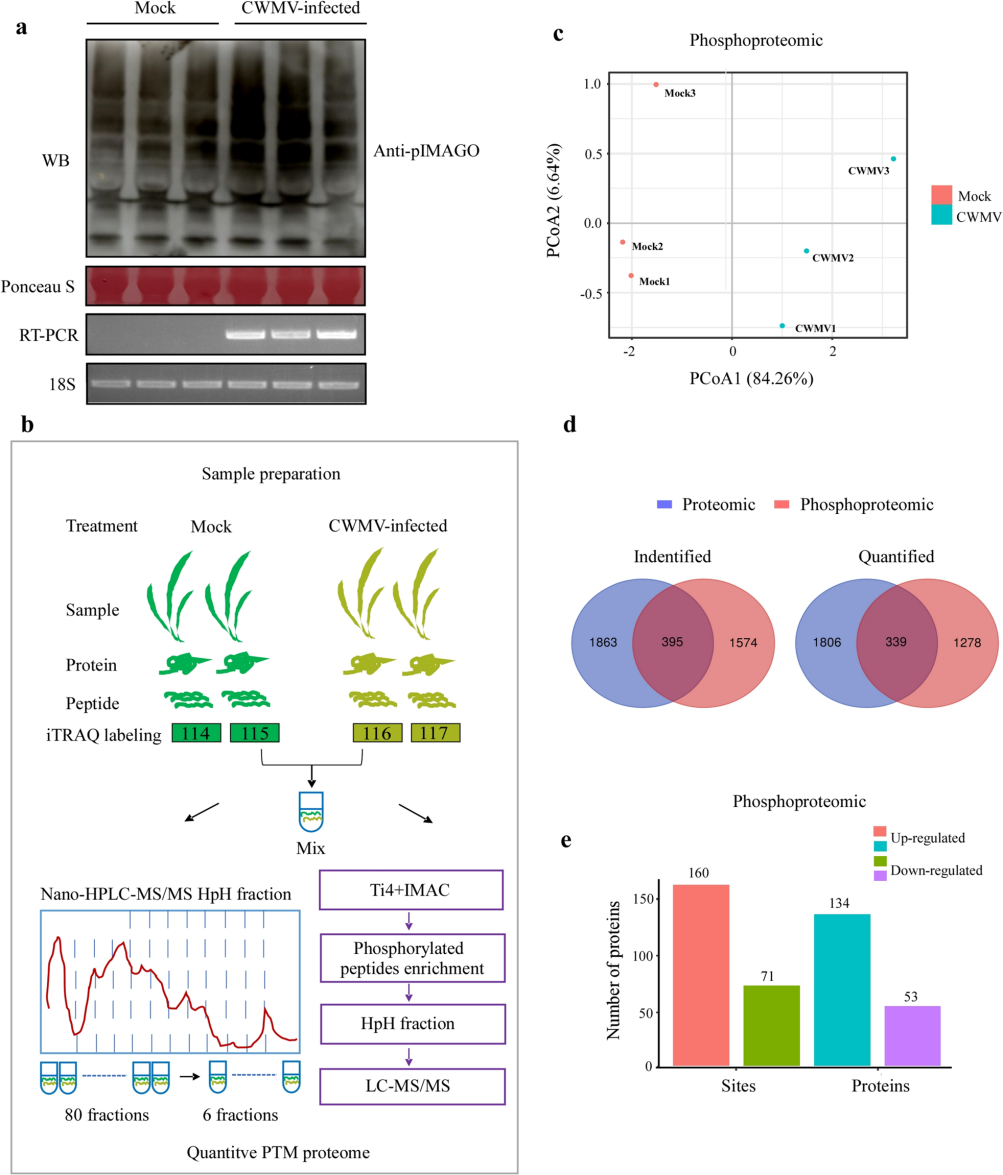
Large-scale proteomics and phosphoproteomics analyses using CWMV-infected (CWMV) and mock-inoculated (Mock) wheat seedlings. **a** Analysis of phosphorylation levels in the Mock (lane 1–3) and CWMV-infected (lane 4–6) wheat seedlings through Western blot assay using an anti-pIMAGO antibody. RT-PCR was performed to confirm CWMV infection using the *CP* gene specific primers. 18S and Ponceau S-stained gel was used to show sample loadings. Full-length blots are presented in Fig. S2-1a. **b** The experimental workflow for the phosphoproteomics analysis is shown. **c** The principal component analysis (PCA) score plot is presented using the LC/MS data. **d** Venn diagrams are used to show the overlaps between the proteomics and phosphoproteomics data using the identified and quantified phosphorylated proteins. **e** Differential expressions of the phosphorylated proteins and sites in the CWMV-infected wheat seedlings are shown

Large-scale phosphoproteomic analysis was performed using three CWMV-inoculated wheat seedlings and three mock-inoculated (control or Mock) wheat seedlings. These samples were first analyzed for CWMV infection through Western blot assays using a CWMV CP specific antibody. Global phosphorylation changes in the assayed seedlings were then analyzed through Western blot assays using an anti-pIMAGO antibody. The results showed that the levels of protein phosphorylation in the CWMV-infected wheat seedling were significantly increased (Fig. 1a).

To further identify the proteins that can affect wheat response to CWMV infection, we performed phosphoproteomic identification assays using the Mock and the CWMV-infected samples. The resulting phosphoproteomic data were normalized against the abundance values of the corresponding proteins. An overview of the phosphoproteomic identification assay workflow is presented in Fig. 1b. The principal component analysis (PCA) results agreed with the LC–MS/MS results (Fig. 1c). Further analysis of the proteome data identified 2268 proteins of which 2144 were quantified **(**Table 1**)**. Based on a threshold of 1.5-fold change at *p* < 0.05 thresholds, the expression of 70 phosphorylated proteins were up-regulated, and 7 were down-regulated in the infected seedlings **(**Table 2**)**. A comparison of the phosphoproteomic data identified 5543 phosphorylation sites in 4520 phosphorylated peptides belonging to 2119 phosphorylated proteins **(**Table 1, Table S1**)**. We have also identified 125 novel proteins and 502 novel phosphorylated proteins, indicating that without the enrichment step, some low abundant proteins may not be identified through phosphoproteomic identification assays (Fig. 1d). CWMV infection resulted in a large number of phosphorylated proteins and phosphorylation sites, and 187 phosphorylated proteins and 231 phosphorylation sites showed highly dynamic responses (Table 2, Table S2). For example, after CWMV infection, a total of 160 phosphorylation sites in 134 proteins were phosphorylated, while 71 phosphorylation sites in 53 proteins were downregulated (Fig. 1e).

**Table 1:** Overviews of proteins and Phos sites identified and quantified through proteomics

		**Identified**	**Quantified**
Proteome	Proteins	2268	2144
Phosphoproteome	Phos sites	5543	4095
	Proteins	2119	1617

**Table 2:** Overviews of regulated proteins and Phos sites in the CWMV-infected plants

	**Regulation Types**	**With > 1.5 Fold Changes**
Proteome	Up-regulated ^#^ Down-regulated	70 proteins 7 proteins
Normalized phosphoproteomics	Up-regulated Down-regulated	160 sites 71 sites

## Characterization of phosphorylated peptides

**Fig. 2:**
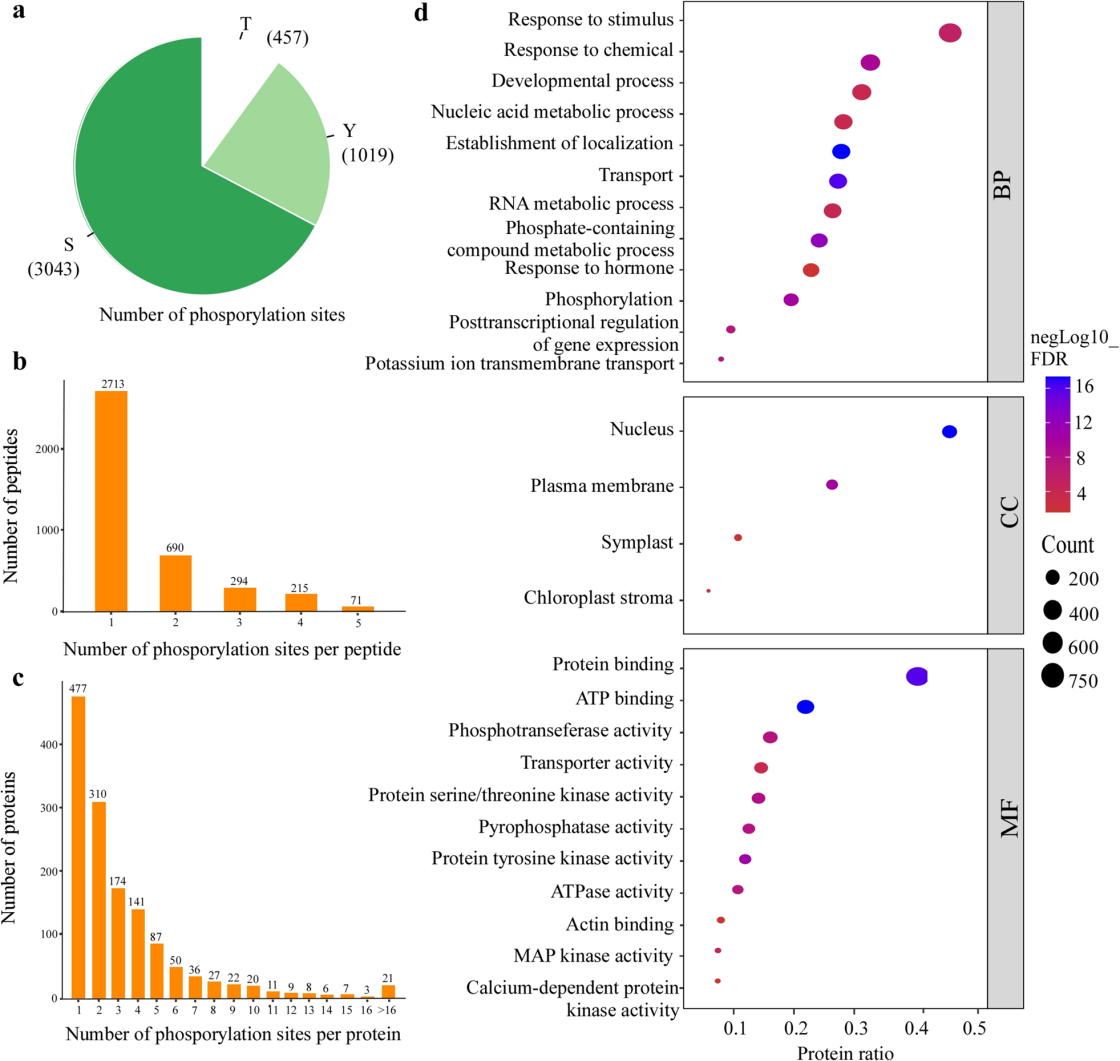
Overview of phosphoproteomic changes in CWMV-infected wheat seedling leaves. **a** Pie chart showing the numbers of phosphorylated serine (pS), phosphorylated threonine (pT), and phosphorylated tyrosine (pY) sites found in this study. **b** Number of phosphorylation sites per peptide. **c** Numbers of proteins with different numbers of phosphorylation sites. **d** GO enrichment result showing phosphorylated proteins identified in this study. BP, Biological Process; MF, Molecular Function; CC, Cellular Component. Significantly enriched terms (adjusted FDR ≤ 0.05) are shown

The numbers of phosphorylation sites in each phosphorylated peptide or protein vary greatly. Serine (S) was the most abundant phosphorylation site (3043, 67.3%), followed by threonine (T) (1019, 22.5%), and then tyrosine (Y) (457, 10.2%) (Fig. 2a). In addition, the phosphorylation sites in peptides with different lengths were unevenly distributed, with approximately 2713 (35.32%) peptides containing only one phosphorylation site (Fig. 2b). Among the phosphorylated proteins, 787 proteins (55.9%) had more than two phosphorylation sites and 21 had more than 16 (Fig. 2c). To investigate the phosphorylation-associated pathways affected by CWMV infection, a Gene Ontology (GO) enrichment analysis was done using 1698 phosphorylated proteins (Fig. 2d). Twelve of them significantly enriched the Biological Processes (BP) terms, including the terms involved in RNA metabolic process, phosphorylation, and post-transcriptional regulations of gene expressions. The ratios of the phosphorylated proteins involved in chemical, developmental process, or establishment of location and transport were relatively greater, suggesting that protein phosphorylation plays a crucial role in CWMV infection in wheat.

### Gene Ontology Pathway Enrichment and subcellular localization analyses using the identified PSPCs

**Fig. 3:**
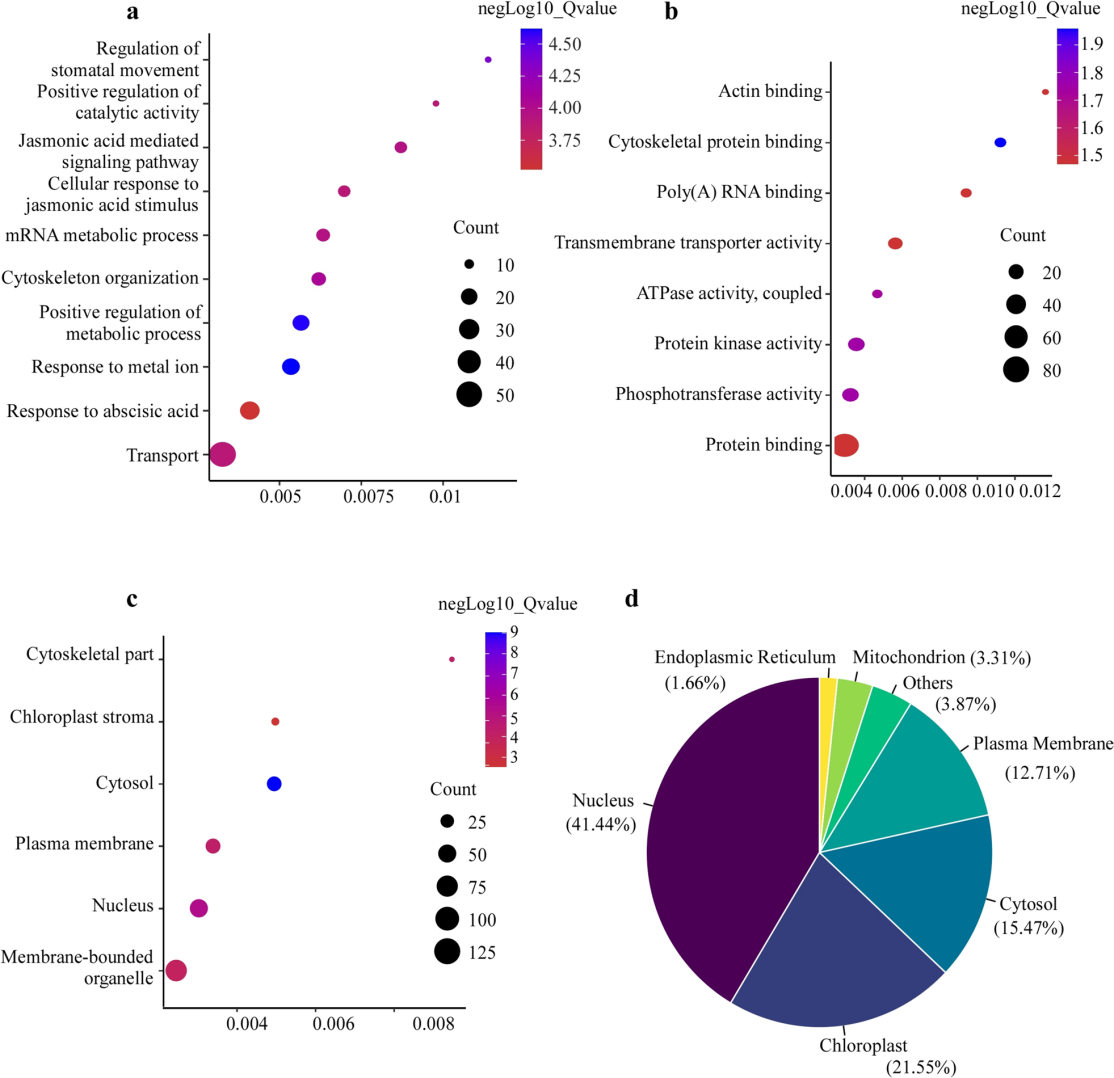
GO enrichment of phosphorylated proteins with significant changes (PSPCs). The phosphorylated proteins were significantly enriched in the biological processes **b** molecular functions and **c** cellular components. The rich factor is the ratio of PSPCs annotated in a pathway term to all the proteins annotated in the same pathway term. The Q-value is the corrected *p*-value ranged from 0 to 1, and a lower *p*-value denotes greater intensity. Circles represent the numbers of enriched genes, and larger circles indicate more enriched genes. **d** Subcellular localizations of all the identified phosphorylated proteins

Protein phosphorylation modification is highly complex. To identify the proteins that are regulated by CWMV infection in wheat, we screened phosphorylated peptides with significant changes (SCPPs) using a stringent cutoff criterion (> 1.5 or <  − 1.5-fold change). Through this screen, a total of 231 SCPPs belonging to 187 PSPCs were found. The GO enrichment analysis was then performed using the PSPCs enriched 11 BP terms and 4 CC terms shown in Fig. 3a-3b. The BP terms related to cytoskeleton organization, jasmonic acid mediated signaling pathway, and regulation of stomatal movement, respectively, were affected upon CWMV infection. In the cellular component category, most enriched phosphorylated proteins were found to be associate with cytosol, nucleus, plasma membrane, and cytoskeletal part. The most significantly enriched molecular functions were mainly protein binding, protein kinase activity, and active transmembrane transporter activity (Fig. 3c). GO analysis result indicated that protein phosphorylation was involved in many biological processes and regulated by CWMV infection. It is well known that protein subcellular location is affected by protein phosphorylation. In this study, most phosphorylated proteins were found in associate with the nucleus (41.44%), chloroplast (21.55%), cytosol (15.47%), and plasma membrane (12.71%), respectively (Fig. 3d).

### Kyoto Encyclopedia of Genes and Genomes (KEGG) pathway enrichment and Protein–Protein Interaction (PPI) network analyses

**Fig. 4:**
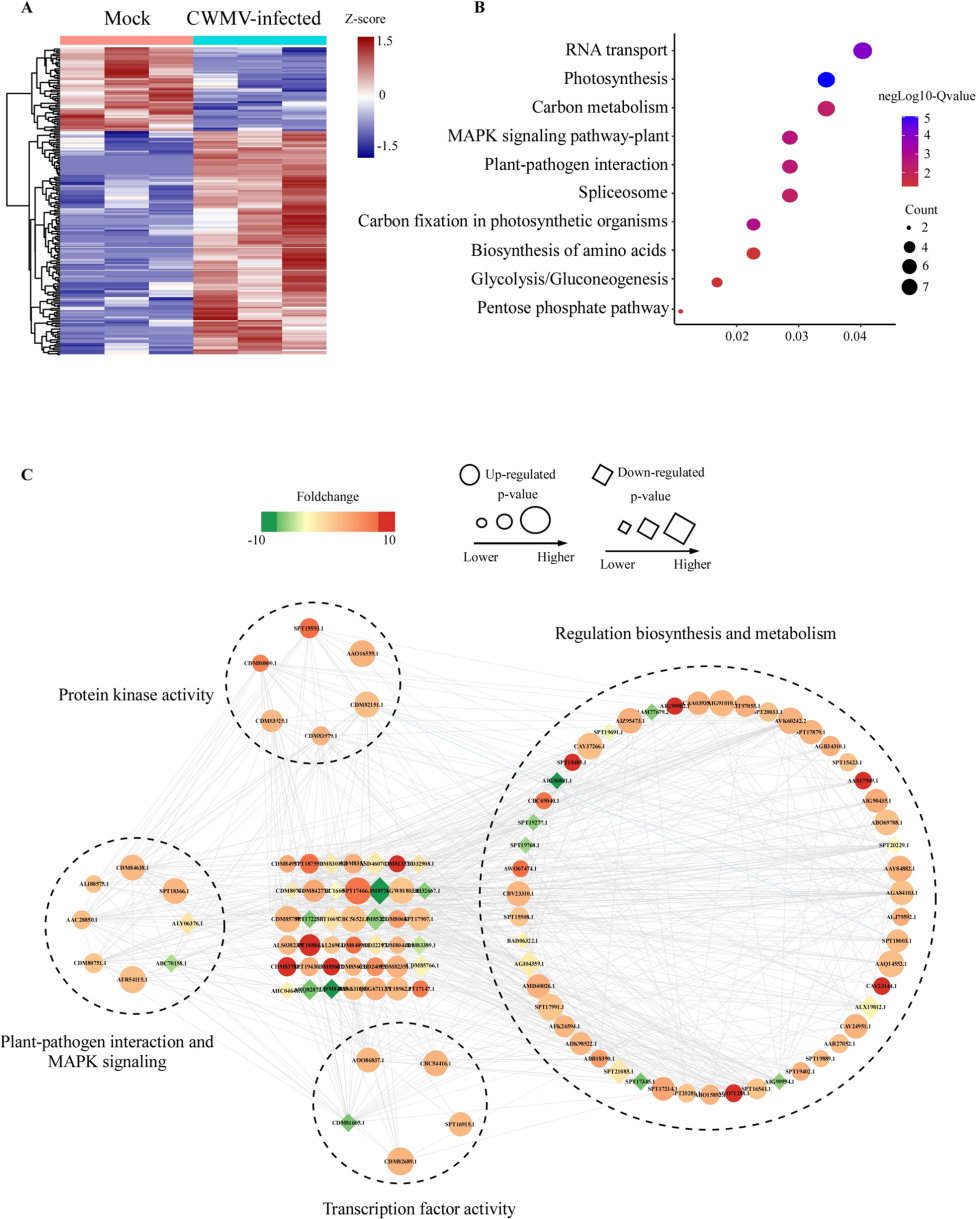
KEGG pathway and interaction network analyses of the phosphorylated proteins with significant changes (PSPCs) in CWMV-infected wheat. **a** Heatmap shows hierarchical clustering of differentially expressed phosphorylated proteins. All relative abundances are row z-score-normalized for visualization. **b** KEGG pathway enrichment analysis of the differentially expressed phosphorylated proteins. **c** Interaction networks for all the identified differentially expressed phosphorylated proteins in CWMV-infected wheat. The Q-value is the corrected *p*-value ranged from 0 to 1, and a lower *p*-value denotes greater intensity. Circles represent the numbers of enriched genes, and larger circles indicate more enriched genes

To investigate the accumulation patterns of the PSPCs regulated by CWMV infection, we performed hierarchical clustering analysis using the average fold change of the intensity ratios normalized against the protein abundance, The phosphorylation levels of 136 proteins (72.7% of the 187 proteins) were significantly increased after CWMV infection (Fig. 4a). To better understand this result; we conducted a functional enrichment analysis to elucidate the Kyoto Encyclopedia of Genes and Genomes (KEGG) pathways using the identified phosphorylated proteins. The significantly enriched pathways, including RNA transport, photosynthesis, and carbon metabolism, were then selected for further analyses (Fig. 4b). The KEGG analysis result also indicated that protein phosphorylation can affect plant responses to CWMV infection by affecting the essential protein biosynthesis and metabolic pathways, including the MAPK signaling pathway and plant-pathogen interaction pathway. To further investigate the significance of protein phosphorylation in CWMV infection, a protein–protein interaction network analysis was performed using the STRING database and the visualization Cytoscape software [31]. Multiple sub-networks, including those regulating biosynthesis and metabolism, protein kinase activity, transcription factor activity, and response to stimuli proteins, were identified using the MCODE software (Fig. 4c). In addition to the plant-pathogen interaction and MAPK signaling-associated proteins, some phosphorylated proteins were found to relate to protein kinase and transcription factor activities, that are widely present in various sub- networks. Therefore, we propose that protein phosphorylation functions at different steps of the host defense cascade by increasing or decreasing the activities of the phosphorylated kinases and transcription factors upon CWMV infection.

### Protein kinase and transcription factor analysis

**Fig. 5:**
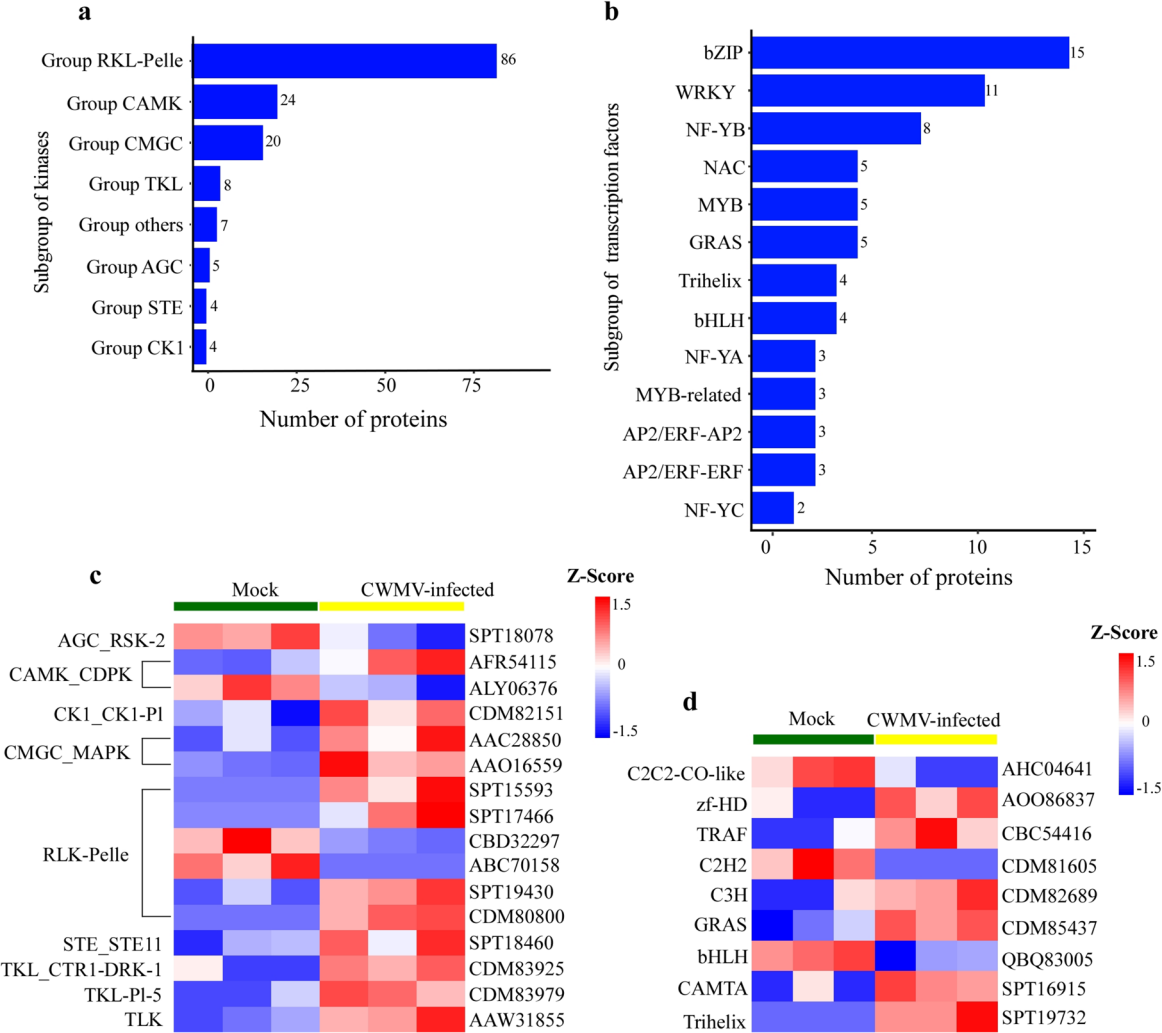
Classification and dynamics of the identified kinases and transcription factors (TFs) in CWMV-infected wheat plants. The identified protein kinases **a** and TFs **b** are classified according to the classification criteria described by Lehti-Shiu and Shiu [32]. **c** A heat map showing kinase activities found in the mock-inoculated (Mock) and CWMV-infected (CWMV) wheat plants, respectively. Columns represent individual samples and rows represent the identified kinases (rows are clustered and Z-scores are normalized). **d** Heat map showing transcription factors found in the Mock and CWMV wheat plants. Columns represent individual samples and rows represent the identified transcription factors (rows are clustered and Z-scores are normalized)

Protein kinases (PKs) are well known to play crucial roles in protein phosphorylation. Although the genes encoding PKs account for only 2% of the genome size in most eukaryotes, they can phosphorylate over 40% of the cellular proteins. Therefore, they are crucial for cellular signaling pathways. To investigate which PK or PKs can regulate protein phosphorylation in CWMV-infected wheat plants, we screened kinases identified in this study. The result showed that the 158 identified kinases could be classified into more than 8 groups (Fig. 5a, Table S3), according to the kinase classification criteria reported by LehtiShiu and Shiu [32]. Among these kinases, 11 were calcium-dependent protein kinases (CDPKs), nine were cyclin-dependent kinases (CDKs), and 8 were mitogen-activated protein kinases (MAPKs). Additionally, 19 kinases were leucine-rich repeat (LRR) proteins that are signature innate immunity-responsive proteins and are involved in the pathogen recognition receptor (PRR) sensing pathways. Among these identified protein kinases, 16 kinases responded to CWMV infection. The phosphorylation levels of most kinases (six RLK-Pelle group kinases, two MAPK cascade pathway-associated kinases, one CDPK, and one tousled-like protein kinase) were increased upon CWMV infection (Fig. 5b, Table S3).

The 71 identified transcription factors (TFs) were classified into 13 groups, including bZIP, WRKY, MYB, and bHLH (Fig. 5c, Table S4). Among these TFs, the phosphorylation levels of seven TFs were significantly altered after CWMV infection, suggesting that the TFs with elevated phosphorylation levels may show higher enzymatic activities during CWMV infection in wheat plants (Fig. 5d, Table S4).

### Phosphorylation dynamics of the two identified defense-related proteins in CWMV-infected plants

**Fig. 6:**
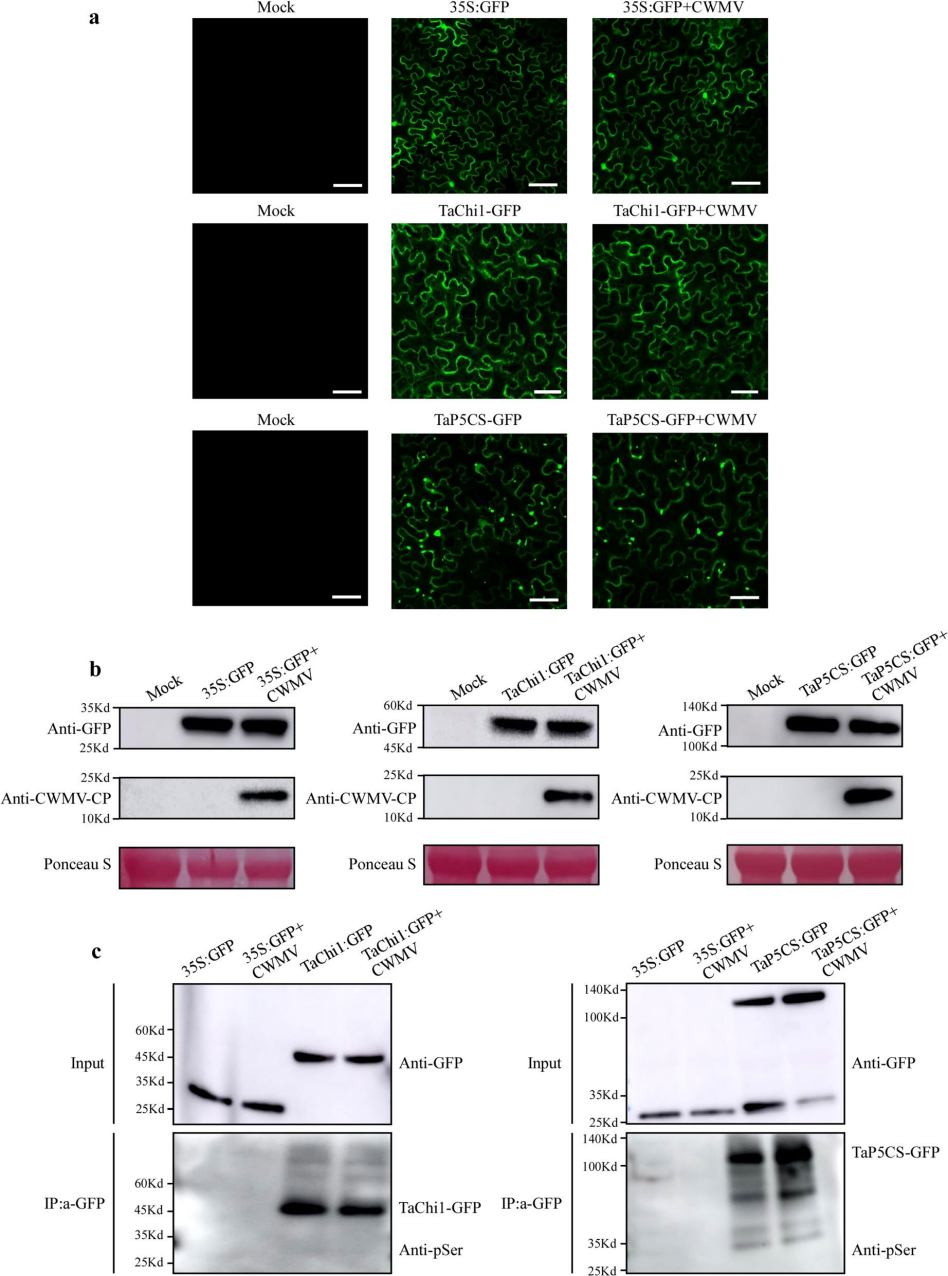
Verification of candidate protein phosphorylation in *N. benthamiana*. **a** Fluorescence intensity in the infiltrated leaves were captured under a confocal microscope. GFP-tagged candidate proteins (TaChi1: GFP and TaChi1: GFP + CWMV) and (TaP5CS: GFP and TaP5CS: GFP + CWMV) were expressed in *N. benthamiana* leaves through agroinfiltration. GFP-tagged candidate proteins (GFP and GFP + CWMV) were expressed in *N. benthamiana* leaves through agroinfiltration as control. Mock treatment represents as the negative control (without GFP). **b** The accumulations of proteins in the leaves were determined through Western blot assay The left group was mock (without GFP) and GFP-tagged proteins (GFP and GFP + CWMV). The middle group was mock (without GFP) and GFP-tagged candidate proteins (TaChi1: GFP and TaChi1: GFP + CWMV). The right group was mock (without GFP) and GFP-tagged candidate proteins (TaP5CS: GFP and TaP5CS: GFP + CWMV). The accumulation of CWMV CP in the assayed leaves was also detected using a CWMV CP specific antibody. Ponceau S-stained gels were used to show sample loadings. Full-length blots are presented in Fig. S2-6b. **c** In vivo phosphorylation assay of TaChi1 and TaP5CS. The left group proteins were extracted from *N. benthamiana* leaves expressing GFP, GFP + CWMV, TaChi1: GFP and TaChi1: GFP + CWMV, respectively. The right group proteins were extracted from *N. benthamiana* leaves expressing GFP, GFP + CWMV, TaP5CS: GFP and TaP5CS: GFP + CWMV, respectively. The proteins were immunoprecipitated with GFP-Trap agarose beads (IP: α-GFP). The phosphorylated proteins were detected through Western blot assays using an anti-pSer antibody. The input was shown in anti-GFP immunoblots. Full-length blots are presented in Supplementary Fig. S2-6c

The integrated phosphoproteomics results obtained through GO functional annotation, KEGG pathway enrichment, and PPI network analyses showed that many of the identified proteins could interact with TaChi1 and TaP5CS (Table S5), suggesting that the phosphorylation levels of the two proteins are affected by CWMV infection. Therefore, the functions of TaChi1 and TaP5CS were further analyzed via protein phosphorylation assays. Full-length *TaChi1* and *TaP5CS* coding sequences were individually amplified, fused with a *green fluorescent protein* gene. The plasmids encoding TaChi1: GFP, TaP5CS: GFP, or GFP alone were transiently expressed in *N. benthamiana* leaves and then examined under a confocal microscope. There was no fluorescence in the mock (without GFP) treatment in either control or experimental groups and no bands were detected by Western-blot assays. The fluorescence from TaChi1: GFP or TaP5CS: GFP fusion was not changed significantly upon CWMV infection (Fig. 6a). This finding agreed with the results of Western–blot assays (Fig. 6b**)**.

To analyze the phosphorylation of TaChi1 and TaP5CS in the CWMV-infected leaves, total proteins were extracted from *N. benthamiana* leaves expressing GFP, TaChi1: GFP, TaP5CS: GFP, GFP + CWMV, TaChi1: GFP + CWMV or TaP5CS: GFP + CWMV, and enriched using GFP-Trap beads. The phosphorylation levels of the proteins were detected through Western blot assays using an anti-GFP or an anti-pSer antibody. The phosphorylation level of TaP5CS was significantly greater in the CWMV-infected plants than in the control (Fig. 6c) whereas the phosphorylation level of TaChi1 in the CWMV-infected plants were significantly decreased upon CWMV infection. Thus, CWMV infection significantly alters the phosphorylation levels of TaChi1 and TaP5CS.

### TaChi1 and TaP5CS can affect wheat defense against CWMV infection

**Fig. 7:**
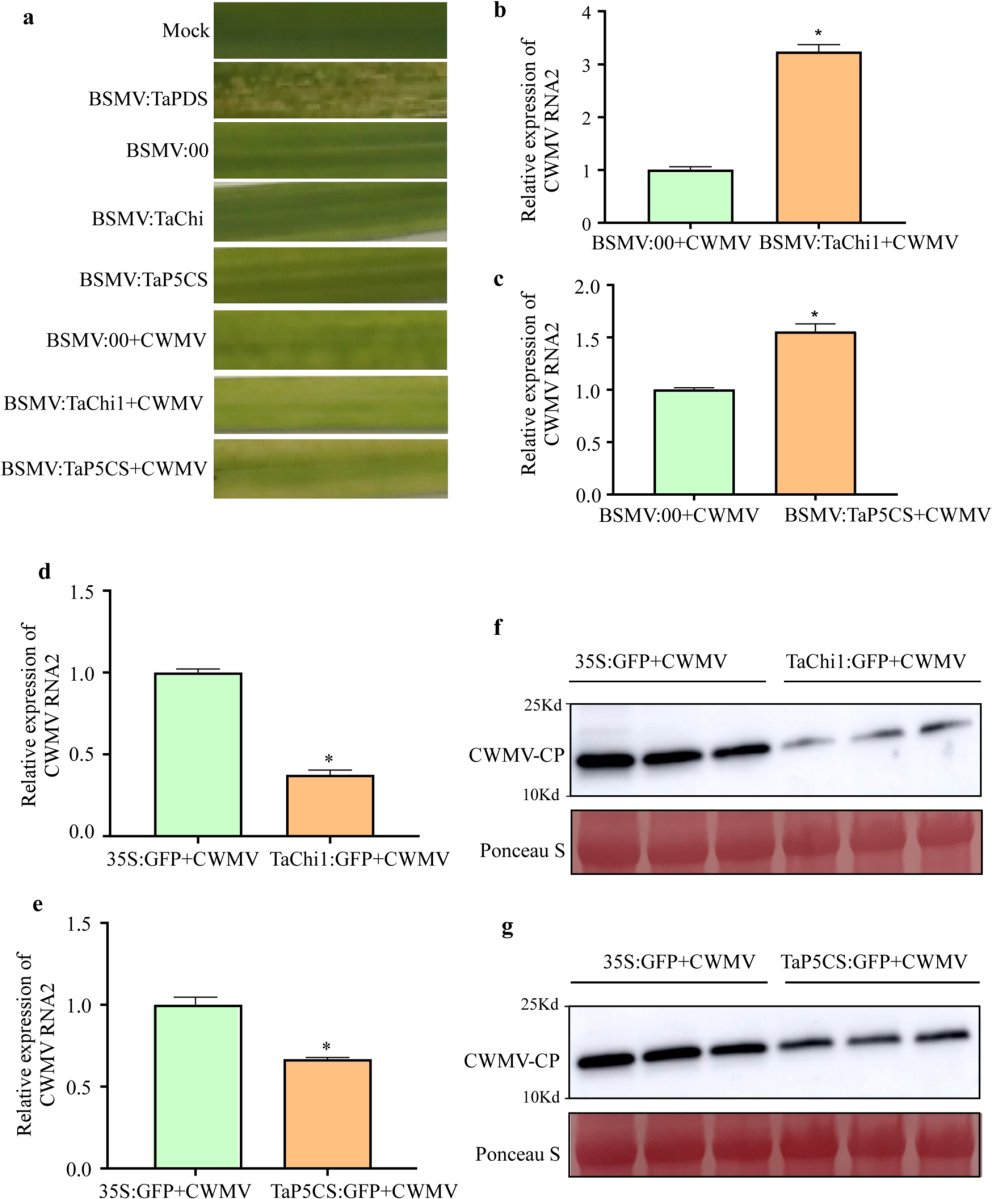
Effects of TaChi1 and TaP5CS on host defense against CWMV infection. **a** Mosaic symptom in the leaves of wheat plants infected with CWMV, BSMV: 00, BSMV: PDS, BSMV: TaChi1, BSMV: TaP5CS, BSMV: 00 + CWMV, BSMV: TaChi1 + CWMV or BSMV: TaP5CS + CWMV. Wheat plants inoculated with FES buffer alone (Mock) were used as controls. The leaves were photographed at 10 days post CWMV inoculation (dpi). **b** Analysis of CWMV RNA2 accumulation in the leaves of the BSMV: 00 + CWMV- or BSMV: TaChi1 + CWMV-inoculated wheat plants through qRT-PCR using CWMV *CP* gene specific primers at 21dpi. The data presented are the means ± SD, determined using the Student’s *t*-test. Each treatment had three biological replicates. *, *P* < 0.05. **c** Analysis of CWMV RNA2 accumulation in the leaves of the BSMV: 00 + CWMV- or BSMV: TaP5CS + CWMV-inoculated wheat plants through qRT-PCR at 21dpi. The data presented are the means ± SD, determined using the Student’s *t*-test. Each treatment had three biological replicates. *, *P* < 0.05. The accumulation levels of CWMV RNA2 in the *TaChi1* or *TaP5CS* transiently overexpressed and CWMV inoculated plants through qRT-PCR using CWMV *CP* gene specific primers at 21 dpi (**d, e**). The accumulation levels of CWMV CP in these assayed plants were determined through Western –blot analysis. (**f**, **g**) GFP-tagged candidate proteins (TaChi1: GFP and TaP5CS: GFP) were transiently co-expressed with CWMV in *N. benthamiana* leaves through agroinfiltration. The GFP + CWMV plants were used as the controls. * Significantly different between groups (*p* < 0.05). Each experiment was repeated twice. The Ponceau S-stained gels were used to show sample loadings. Full-length blots are presented in Figure S2-7f and g

Virus-Induced Gene Silencing (VIGS) using a Barely stripe mosaic virus (BSMV)-based gene silencing vector was done to investigate the roles of TaChi1 and TaP5CS in wheat defense against CWMV infection. The BSMV: TaChi1, BSMV: TaP5CS, and BSMV: 00 (control) viruses were inoculated separately to wheat leaves. Quantitative RT-PCR analyses were performed to check the gene silencing results using primers specific for *TaChi1* or *TaP5CS*. Compared to the BSMV:00-infected wheat plants, the BSMV: TaChi1 or BSMV: TaP5CS-inoculated plants showed a significant reduction (about 50%) of *TaChi1* (Fig. S1a) and *TaP5CS* **(**Fig. S1b) transcripts at 10 days post vector inoculation. These assayed wheat plants were then inoculated with CWMV. At 21 days post CWMV inoculation, stronger mosaic symptoms developed in the leaves of the BSMV: 00 + CWMV-inoculated plants than that in the leaves of the BSMV: TaChi1 + CWMV- or BSMV: TaP5CS + CWMV-inoculated plants **(**Fig. 7a**)**. The results of qRT-PCR using the CWMV *CP* gene-specific primers showed that the accumulation level of CWMV RNA2 in the BSMV: TaChi1 + CWMV- or the BSMV: TaP5CS + CWMV-inoculated plants was significantly increased **(**Fig. 7b-c**)**.

Because CWMV can infect the model *N. benthamiana* plants, we also analyzed CWMV CP expression in *N. benthamiana* leaves at 5dpi agroinfiltration through qRT-PCR and Western blot assays. In the presence of CWMV, the expression levels of *TaChi1* and *TaP5CS* were reduced (Fig. 7d-g**)**, indicating that the expressions and functions of TaChi1 and TaP5CS can be affected by CWMV infection in plants.

## Discussion

### Phosphorylation was essential during CWMV infection

During crop production, many abiotic and biotic stress factors, including pathogens, can seriously damage crop growth and yield [21]. Protein phosphorylation is a well-known post-translational process that modifies proteins to enhance or suppress their cellular functions, including their response to virus infections [33]. In this study, we analyzed the wheat response to CWMV infection using a large-scale phosphoproteomic analysis. Through this study, a total of 5543 phosphorylation sites were found in 4520 phosphorylated peptides belonging to 2119 proteins (Fig. 1d**)**. This suggests that changes in phosphorylation occur widely during CWMV infection. Similar studies have been conducted using *A. thaliana*, rice, maize, and *Brachypodium distachyon* [34–37]. For example, Xing and others reported a comprehensive phosphoproteomics analysis using cold-stressed maize seedlings, and identified 19,320 phosphopeptides in 4803 proteins [38]. In this study, a total of 1102 phosphorylated sites in 962 phosphorylated proteins were found to match that reported for the drought-stressed wheat plants [39]. In 2015, Hou and others reported 2367 and 2223 phosphosites in 1334 and 1297 proteins at 0 h and 24 h respectively after infection by *Xanthomonas oryzae pv.oryzae* (*Xoo*) infection [7]. In our study, these identified phosphorylated proteins were predicted to participate in wheat growth, responses to various stimuli and hormones, and many biosynthesis and metabolism pathways (Fig. 2d**)**. Some of them are well-known pathogen response phosphoproteins but most of the others have not been reported to be involved with virus stress. Furthermore, some phosphoproteins were regulated following abiotic stress in other plants. This suggests that plants response to abiotic or biotic stresses are not independent but participate in crosstalk with one another in networks [40–42]. These reports indicate that protein phosphorylation commonly occurs in stressed plants and is important in host defense responses.

### Protein kinase cascades are widely involved in CWMV infection

Phosphorylation and dephosphorylation of proteins are important regulatory processes and can change substrate activities. In this study, we have identified 231 SCPPs that matched 187 PSPCs in the CWMV-infected wheat plants (Fig. 1e**)**. Because many phosphorylated proteins can be dephosphorylated, the plant can utilize complex signaling cascades to defend against pathogen infections [43]. Plant genome have more kinase genes (940 in Arabidopsis) than phosphatase genes (150 in Arabidopsis), suggesting that the substrate specificity is higher for kinases than for phosphatases [2]. Among these kinases, the receptor-like kinases (RLKs), receptor-like cytoplasmic kinases (RLCKs), and calcium-dependent protein kinases (CDPKs or CPKs) are known to be the key regulators of plant immunity [44–46]. In this study, 11 calcium-dependent protein kinases (CDPKs), nine cyclin-dependent kinases (CDKs), eight mitogen-activated protein kinases (MAPKs), and 19 leucine-rich repeat (LRR) proteins were found to be phosphorylated upon CWMV infection (Fig. 5a). TaCDPK7-D is one down-phosphorylated CDPK we identified. *TaCDPK7-D* has been shown to positively regulate host resistance to eyespot disease, probably by regulating the expression of defense-associated and ethylene biosynthesis genes [47]. We hypothesized that the downregulation of TaCDPK7-D phosphorylation during the CWMV infection might result in this positive regulator being in an "inactive" state, thus favoring virus infection.

Many protein kinases have multiple phosphorylation sites, and the phosphorylated kinases can sense and integrate the information generated by different signaling pathways. These reports suggest that host plants rapidly alter kinase activities through phosphorylation in response to pathogens. Some of these protein kinases are known to be related to biotic or abiotic stress and participate in crosstalk with one another, while at the same time playing different roles in response to complex environmental stresses. Our GO and KEGG enrichment analysis results revealed that many phosphorylated proteins are involved in the MAPK signaling pathway, response to hormone stimuli, and plant-pathogen interactions (Fig. 3a-3c, 4b**)**. Our results are consistent with previously published results showing that protein phosphorylation is important in the response of maize to Sugarcane mosaic virus infection, and the phosphorylated proteins identified in that study were also enriched in the plant-pathogen interaction category and the MAPK signaling pathway [48]. In 2021, He and others identified 390 differentially expressed proteins (DEPs) in the CWMV-infected *N. benthamiana* plants, and these DEPs were also enriched in the MAPK signaling pathway [49]. The signals generated by receptor kinases (from MAPKKK to MAPKK and then MAPK) can be transmitted and amplified to reprogram host cellular responses to stimulus [50]. MAPKs are the last component of a conserved three-kinase module of MAPK cascades [51]. In our study, the phosphorylation levels of two MAPK homologues were significantly increased. Unlike previous studies, OsMAPK6, a key component in the OsRac1-OsMAPK3/6-RAI1-PAL1/OsWRKY19 rice immunity signaling cascade, was down-phosphorylated at 24 h of Bacterial blight inoculation. It suggests that, unlike the classical immunity signaling cascade, MAPK kinases play different roles in response to different pathogens during the arms race between plant and pathogen.

### Phosphorylation of transcription factors play important roles during CWMV infection

Through the kinase cascade controlled by kinase or phosphatase, the signals of pathogen infection stimuli could be transmitted to the nucleus and disease resistant-associated proteins are then directly or indirectly phosphorylated or dephosphorylated to initiate immune responses. In Tobacco mosaic virus-infected plants, a total of 337 phosphopeptides, belonging to 277 different phosphoproteins, were found to be protein kinases, phosphatases, and translation initiation or transcription-associated factors [52]. In recent studies, many TFs have been found to serve as important internodes in disease resistance-associated pathways by linking MAPK signals with downstream transcriptional reprogramming [53, 54]. In this study, at least 71 TFs consisting of bZIP, WRKY, MYB and bHLH family members, were phosphorylated. STRING analysis results showed that the identified biosynthesis and metabolism-associated proteins, PKs, and TFs were important for protein–protein interactions in the CWMV-infected wheat plants (Fig. 4c.) Our subcellular localization analysis result showed that 41.44% of the identified phosphorylated proteins were localized in the nucleus (Fig. 3d**)** and the phosphorylation levels of nine TFs were significantly changed (Fig. 1e**)**. It is worth noting that we identified a reported MYC4 transcription factor that positively regulates the jasmonic pathway. JAs are important signaling molecules within plant cells, regulating the expression of defense proteins and the synthesis of secondary metabolites through interactions with transcription factors [55]. MYC4 is a JAZ-interacting transcription factor that act together with MYC2 and MYC3 to regulate JA-responses [56, 57]. We hypothesize that the significantly increased phosphorylation of MYC4 after CWMV infection might be due to the production of large amounts of jasmonic acid in the host, which deregulates the inhibitory effect of MYC2 and activates the transcriptional activity of MYC4 to initiate the transcription of JA-responsive genes. It suggests that the phosphorylated proteins can sense and integrate the information generated by different signaling pathways including hormone synthesis and metabolism.

### Phosphorylation of TaChi1 and TaP5CS is important to wheat resistance to CWMV infection

Plant response to invading pathogens is a complex process determined by the physiological reactions mediated by enzyme-catalyzed activities. Phosphorylation and dephosphorylation of specific sites in the target proteins can yield modified proteins with new functions or structural stabilities [7]. In this study, the phosphorylated peptides were found to be enriched in the positive regulation of catalytic process terms following CWMV infection (Fig. 3a). The protein–protein interaction analysis result showed that many identified phosphorylated proteins are involved in the regulations of biosynthesis and metabolism (Fig. 4c). These enrichments are also closely related to enzyme catalytic process. It is known that that many defense-associated enzymes can scavenge reactive oxygen species (ROS) or synthesize defense-related substances, such as phenols, lignans, and phytochemicals, to enhance plant resistance to diseases [58]. In our study, two enzymes related to plant immunity were enriched in the positive regulation of catalytic process and plant-pathogen interaction pathway. Transient expression of TaChi1 or TaP5CS in *N. benthamiana* was not significantly affected by CWMV infection (Fig. 6a, b), implying that phosphorylation may be an important step for activation of pre-existing TaChi1and TaP5CS during CWMV infection.

Chitin is a vital building block of fungal cell walls and an effective elicitor of plant immunity [59]. Plant chitinases are pathogenesis-related proteins that can participate in plant defense against pathogen infections [60, 61]. On the one hand, plant chitinases can also hydrolyze fungal cell walls to release chitin oligomers, which are subsequently sensed by plant chitin receptor complexes localized near the plasma membrane (PM) to initiate intracellular immune signaling and then chitin responses and fungal resistance [62]. Chitinase activity is known to be regulated by salicylic acid (SA) or jasmonic acid (JA) [63]. In rice, the expressions of *OsChi1a* and *OsChi1c* have been shown to induce transient JA biogenesis. In addition, the application of JA has been shown to increase the activities of several chitinases and to activate the induced systemic resistance (ISR) in plants [64]. In our study, the JA signaling pathway was enriched in the BP terms upon CWMV infection (Fig. 3a). These findings agreed with previous reports that plants can regulate the JA signaling pathway to sense the pathogen-generated stimuli to program their immune response. On the other hand, the results shown in Fig. 6c indicate that CWMV can also reprogram the JA-mediated signaling pathway by decreasing chitinase phosphorylation to suppress ISR.

Proline accumulation is increased in plants under drought, salt, oxidative, or biotic stresses [65]. *P5CS* is known to encode a bifunctional enzyme that can catalyze rate-limiting proline biosynthesis in plants [66]. In 2004, Fabro and colleagues reported that the expression of *P5CS1* was not changed significantly after bacterial infections, whereas the expression of *P5CS2* was upregulated in Arabidopsis during a hypersensitive response to an avirulent *Pseudomonas syringae* strain [67]. A different report has shown that proline can stabilize proteins, membranes, and other subcellular structures during stresses [68]. Our group had previously reported that many immunity-related proteins were degraded in the CWMV-infected wheat plants via the ubiquitin-26S proteasome system [69]. It is noteworthy that the phosphorylation level of TaP5CS was increased after CWMV infection (Fig. 6c**)**. We speculate that the enhanced phosphorylation level of TaP5CS may improve its activity to increase proline biogenesis to prevent host immunity-related proteins from being degraded through the ubiquitin-mediated pathway during CWMV infection.

## Conclusions

CWMV can survive many years in the dormant spores of *P. graminis* in soil, making the disease difficult to control [22, 23]. The use of disease-resistant wheat varieties is the most effective and environmentally friendly control method but, although numerous attempts have been made to screen wheat for CWMV resistance, only a few CWMV-resistant lines or genes have been identified [21]. Through this study, we have obtained a comprehensive phosphoproteomic atlas related to CWMV infection in wheat. Analysis of this atlas identified 187 phosphorylated proteins with significantly altered phosphorylation levels. Of these phosphorylated proteins, many are involved in the positive regulations of catalytic processes, MAPK signaling pathways, protein kinase activities, and transcription factors. This finding indicates that during CWMV infection, protein phosphorylation plays a key role in regulating host defense-related signaling cascades. In addition, we identified two enzymes related to plant immunity and show that the phosphorylation of TaChi1 and TaP5CS plays important roles in wheat resistance to CWMV infection. These results should broaden our understanding of the arms race between wheat and CWMV and benefit future anti-CWMV wheat breeding.

## Methods

### Plant materials and sample preparation

*Nicotiana benthamiana* and CWMV susceptible wheat (*Triticum aestivum*) cv. Yangmai158 were used in this study. Seedlings were grown in a greenhouse maintained at 17℃, 16 h light at 200 µmol m^−2^ s^−1^ and 8 h dark, and 70% relative humidity. Two-leaf-stage wheat seedlings were rub-inoculated with in vitro transcribed CWMV transcripts. Wheat seedlings inoculated with buffer were used as controls. At seven days post inoculation (dpi), the inoculated seedlings were separately harvested, frozen in liquid nitrogen, and then stored in a − 80℃ freezer till further use. Three independent biological replicates were collected from each treatment.

### Protein extraction and digestion

Approximately 2 g tissues were collected from each frozen wheat sample, placed in a pre-cooled grinding tube, and ground into powders in liquid nitrogen. Each powdered sample was transferred into a 5 ml centrifuge tube, four volumes of a lysis buffer [8 M urea, 10 mM dithiothreitol, 1% Triton-100, 3 µM trichostatin A (TSA), 50 mM nicotinamide (NAM), and 1% protease inhibitor cocktail] were added into the tube, and then sonicated three times on ice using a high-intensity ultrasonic processor (Scientz, Ningbo, China). An equal volume of Tris-saturated phenol, pH 7.8, was added into the tube and the mixture was vortexed for 5 min followed by 15 min centrifugation at 10000* g* and 4℃. The upper phenol phase was carefully transferred into a clean centrifuge tube. Four volumes of ammonium sulfate-saturated methanol was added into the tube and the tube was incubated at -20 ℃ for over 6 h to precipitate proteins. After 10 min centrifugation at 4 °C, the pellet was rinsed once with an ice-cold methanol and then three times with an ice-cold acetone. The resulting pellet was dissolved in an 8 M urea and its protein concentration was determined with a BCA kit (Abcam, Cambridge, MA, USA) as instructed. The trypsin was used for protein digestion, according to the description of Ye et al. [35].

### Tandem Mass Tag labeling and phosphopeptide enrichment

The trypsin-digested peptides were labeled with tandem mass tags (TMT) using a TMT kit according to the instruction. Briefly, the trypsin-digested peptides were vacuum-frozen and dried after desalination with the Strata X C18 (Phenomenex, Torrance, CA, USA). The treated peptides were reconstituted in a 0.5 M TEAB solution and further processed using the TMT kit/iTRAQ kit as instructed by the manufacturer. The resulting samples were fractionated through high pH reverse-phase HPLC using the Agilent 300 Extend C18 column (5 μm particles, 4.6 mm ID, 250 mm in length).

The Ti4 + -IMAC microspheres suspensions were individually incubated in a loading buffer containing 80% ACN and 6% TFA, saturated with glutamic acid. The resulting tryptic peptides were resuspended in the loading buffer and mixed with Ti4 + -IMAC microspheres (1:10, w/w) followed by 30 min shaking. The Ti4 + -IMAC microspheres were pelleted through centrifugation, resuspended in a washing buffer (50% ACN, 6% TFA, 200 mM NaCl), and shaken for 30 min (twice). Finally, the Ti4 + -IMAC microspheres were eluted twice with an elution buffer (500 mM NH_4_OH and 60% ACN) and the phosphopeptides were collected and lyophilized prior to LC–MS/MS analysis.

### LC–MS/MS analysis

Enriched and lyophilized peptides were dissolved in the liquid chromatography mobile phase A (0.1% formic acid) and separated through EASY-nLC1000 ultra-high performance liquid chromatography (UPLC). The separated peptides were injected into the NSI ion source followed by tandem mass spectrometry (MS/MS) and then analyzed by Q Exactive ™ Plus mass spectrometer (Thermo Fisher Scientific, Rockford, IL, USA) coupled online to the UPLC system. The peptide segments and their secondary fragments were detected and analyzed using a high-resolution Orbitrap spectrometer (Thermo Fisher Scientific). The selection of peptides for MS/MS was carried out using NCE with a resolution of 17,500. A single MS scan and 20 MS/MS scans were set with a 15.0 s dynamic exclusion. The parameters of the fixed first mass and the automatic gain control were 100 m/z and 5E4, respectively.

### Database search and bioinformatics analysis

The resulting LC–MS data were processed using the MaxQuant search software (v.1.5.2.8). The tandem mass spectra were used to search annotated protein sequences deposited in the *Triticum aestivum* database (http://plants.ensembl.org/Triticum_aestivum/Tools/Blast, accessed on 8 May 2021) using the following parameters: tryptic peptides with up to four missed cleavages; 20 and 5 ppm mass tolerances in the first and the main searches, respectively; and 0.02 Da for the MS/MS fragment ions. Additionally, a false discovery rate (FDR) of 0.01 was used for proteins and peptides, and the minimum score for the modified peptides was set to 40.

Bioinformatics analysis was performed according to the previously described protocols [18, 20, 52, 70]. The obtained proteins were further classified into three categories (biological process, cellular compartment, and molecular function) using the Uniprot-GOA database (http://www.ebi.ac.uk/GOA/, accessed on 20 May 2021) as a reference. Functional annotations of the phosphorylated proteins were performed according to the Kyoto Encyclopedia of Genes and Genomes (KEGG) database (http://www.kegg.jp/kegg/pathway.html, accessed on 15 July 2021) using the KEGG online service tool KEGG Mapper. The two-tailed Fisher’s exact test was then employed to validate the enrichment results of GO terms and KEGG pathways. The enriched GO terms and KEGG pathways with a *P*-value < 0.05 were statistically significant. The PPI networks were constructed using the STRING software (https://string-db.org/, accessed on 21 June 2021) and presented using the Cystoscope software (version 3.8.0).

### Construction of recombinant plasmids

A high-fidelity enzyme was used to amplify the full-length *TaChi1* and *TaPS5C* coding sequences through PCR using specific primer pairs (Table S5). The amplified sequences were cloned individually behind the Lac promoter inside the Pcambia 1305 vector and then transformed into *E. coli*. Three positive *E. coli* clones were selected from each transformation even, sequenced, and transformed individually into *Agrobacterium tumefaciens* strain GV3101.

### Transient gene expression and virus inoculation

To investigate the roles of TaChi and TaPS5C on CWMV infection, a green fluorescent protein (GFP) was fused to the C terminus of TaChi and TaPS5C to produce TaChi1: GFP and TaP5CS: GFP fusions, respectively. The fusion proteins were individually co-expressed with CWMV in *N. benthamiana* leaves via agroinfiltration. The *N. benthamiana* leaves co-expressing 35S: GFP and CWMV were used as controls. At five days post agroinfiltration, the leaves showing green fluorescent under a confocal fluorescence microscope were collected and analyzed for CWMV accumulation and the phosphorylation levels of TaChi: GFP and TaPS5C: GFP.

### RNA extraction and quantitative Reverse Transcription-PCR (qRT-PCR) assays

Total RNA was isolated from the leaves of *N. benthamiana* plants inoculated with CWMV, reverse transcribed (1 μL total RNA per reaction) using the First Stand cDNA Synthesis Kit (Toyobo, Osaka, Japan) followed by quantitative PCR (qPCR) on a 7900 Real-Time PCR machine (Applied Biosystems, Foster City, CA, USA) using the AceQ RT-qPCR SYBR Green Master Mix Kit (Vazyme, Nanjing, China). Each qPCR reaction (20µL) contained 10 µL SYBR Green Master Mix, 2 µL cDNA, 0.5 µL of 10 µM forward and reverse primers, and 7 µL ddH_2_O. The qPCR conditions were as: 95 °C for 3 min, 40 cycles of 95 °C for 30 s and 60 °C for 30 s, and 72 °C for 30 s. Three biological replicates with four technical repeats were analyzed for each treatment. The expression of the β-*Actin* gene was used as the internal reference. The primers used in this assay were listed in the supplementary information (Table S6).

### Western blot analysis of phosphorylated proteins

Expressions of the GFP-fused proteins were monitored under fluorescence microscopy. Fresh leaf tissues (0.1 g per sample) were ground in liquid nitrogen and then lysed in 500µL (per sample) of immunoprecipitation (IP) buffer [150 mM NaCl, 50 mM Tris–HCl (pH 7.5), 5 mM Na_2_EDTA, 10 mM dithiothreitol, 1% (v/v) Triton X-100, 2 mM Na_3_VO_4_, 2 mM NaF,1 mM DTT, and 1% protease inhibitor cocktail]. The crude extracts were transferred into microcentrifuge tubes and centrifuged at 12000* g* for 15 min at 4℃. The GFP-fused proteins were immunoprecipitated using the GFP-Trap agarose beads (Sigma-Aldrich, St. Louis, MO, USA). The protein extracts (400 µL per sample) were individually incubated with 25µL GFP-Trap beads for 3 h at 4℃with gentle shaking. The GFP-trap agarose beads were pelleted and washed three times with a 50 m Tris–HCl, pH 7.5, solution. The bound protein was eluted using an elution buffer (50 mM Tris–HCl, pH 7.5), and the resulting protein samples were mixed with an SDS-PAGE sample buffer followed by 10 min incubation at 100 ◦C. The protein samples were loaded individually into wells of 10% SDS-PAGE gels, separated through electrophoresis, and then transferred to nitrocellulose (NC) membranes. The accumulation level of CWMV and the phosphorylation level of the assayed proteins were determined through Western blot assays using an anti-CWMV CP antibody, a 1:2000 diluted anti-phos-ser rabbit antibody (Abcam, Inc., Atlanta, GA, USA), and a 1:5000 diluted anti-GFP mouse antibody (Abcam). The secondary antibodies used in this study were an anti-rabbit or an anti-mouse antibody conjugated to HRP (1:10,000, Invitrogen, Carlsbad, CA, USA).

### Virus-induced gene silencing assay using a BSMV-based vector

The VIGS assay was done as previously reported [30]. Briefly, a 300-bp fragment was RT-PCR amplified from the coding sequence of *TaChi* or *TaP5CS* and cloned into the pBSMVγvector. Plasmid pBSMVα, pBSMVβ, pBSMVγ, pBSMVγ:TaChi, pBSMVγ:TaP5CS, and pBSMVγ:TaPDS were individually linearized using specific restriction enzymes, and then used to produce in vitro RNA transcripts using a mMessage mMachine T7 transcription kit as instructed (Ambion, Austin, TX, USA). The in vitro transcribed BSMVα transcripts were mixed with that of BSMVβ and that of BSMVγ:TaChi, BSMVγ:TaP5CS, BSMVγ:TaPDS or BSMVγ at 1:1:1 (v/v/v) ratio. The mixed RNA transcripts were rub-inoculated onto the second true leaf of individual wheat seedlings. The seedlings inoculated with the mixed BSMV:00 and BSMV: TaPDS RNA transcripts were used as controls. Ten days later, these plants were inoculated again with sap from CWMV-infected leafs and then grown in a greenhouse maintained at 15℃.

### Supplementary Information

**Additional file 1.** 

** Additional file 2.** 

** Additional file 3.** 

** Additional file 4.** 

** Additional file 5.** 

** Additional file 6.** 

** Additional file 7.** 

** Additional file 8.** 

** Additional file 9.** 

## Data Availability

All data generated or analyzed in this study are included in this article and its supplementary materials.

## Abbreviations

CWMV: *Chinese wheat mosaic virus*
*P. graminis*: *Polymyxa graminis*
LC–MS: Liquid Chromatography-Mass Spectrometry
PTM: Post-translational modification
PTI: Pattern-triggered immunity
ETI: Effector-triggered immunity
PCA: Principal component analysis
PSPCs: Phosphorylated proteins of significant changes
SCPPs: Phosphorylated peptides with significant changes
GO: Gene Ontology
BP: Biological Processes
CC: Cellular Component
MF: Molecular Function
KEGG: Kyoto Encyclopedia of Genes and Genomes
STRING: Search Tool for the Retrieval of Interacting Genes/Proteins
PPI: Protein–protein interaction network analysis
TaP5CS: Delta-1-pyrroline-5-carboxylate synthase in *Triticum aestivum* L
TaChi1: Chitinase1 in *Triticum aestivum* L
