# Supplementary material for: Large-scale phosphoproteome analysis in wheat seedling leaves provides evidence for extensive phosphorylation of regulatory proteins during CWMV infection
Source: BMC Plant Biol. 2023 Nov 2;23:532. doi: 10.1186/s12870-023-04559-3 (PMC10621099; doi:10.1186/s12870-023-04559-3)
Supplement: Supplementary file 1 — Additional file 1. [file 12870_2023_4559_MOESM1_ESM.docx]

**
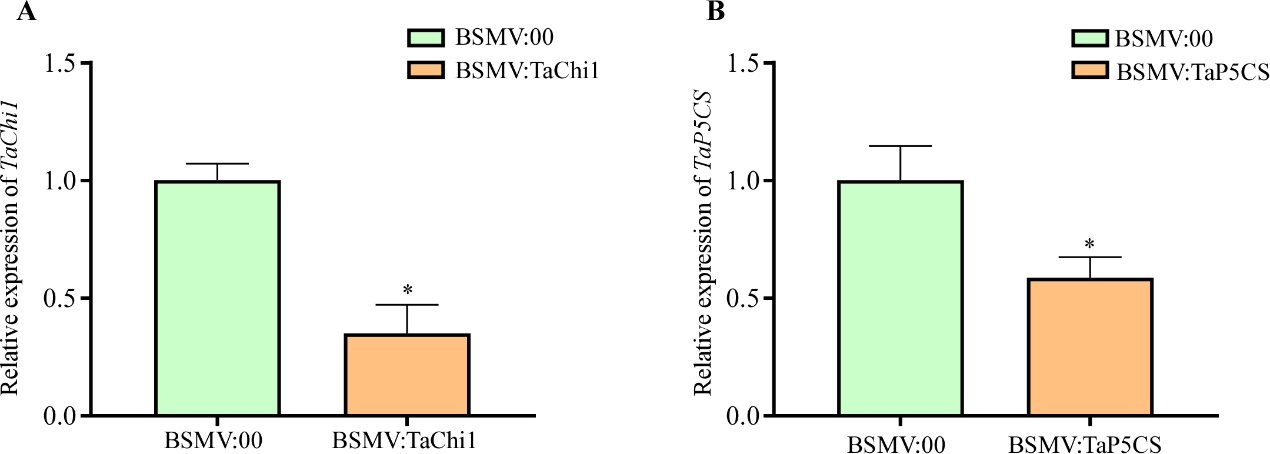
a b**

**Supplement Fig.1a and Fig.1b** **Analyses of *TaChi1* and *TaP5CS* mRNA and protein expressions in wheat plants.** a. Relative expression levels of TaChi1 mRNA in the BSMV:00+CWMV- or BSMV: TaChi1 +CWMV-inoculated wheat plants were determined through qRT-PCR at 10 days post BSMV inoculation. The data presented are the means ± SD, determined using the Student’s *t*-test. Each treatment had three biological replicates. *, P <0.05. b. Relative expression levels of TaP5CS mRNA in the BSMV:00+CWMV- or BSMV: TaP5CS +CWMV-inoculated wheat plants were determined through qRT-PCR at 10 days post BSMV inoculation. The data presented are the means ± SD, determined using the Student’s *t*-test. Each treatment had three biological replicates. *, P <0.05.
