# Supplementary material for: Large-scale phosphoproteome analysis in wheat seedling leaves provides evidence for extensive phosphorylation of regulatory proteins during CWMV infection
Source: BMC Plant Biol. 2023 Nov 2;23:532. doi: 10.1186/s12870-023-04559-3 (PMC10621099; doi:10.1186/s12870-023-04559-3)
Supplement: Supplementary file 2 — Additional file 2. [file 12870_2023_4559_MOESM2_ESM.docx]

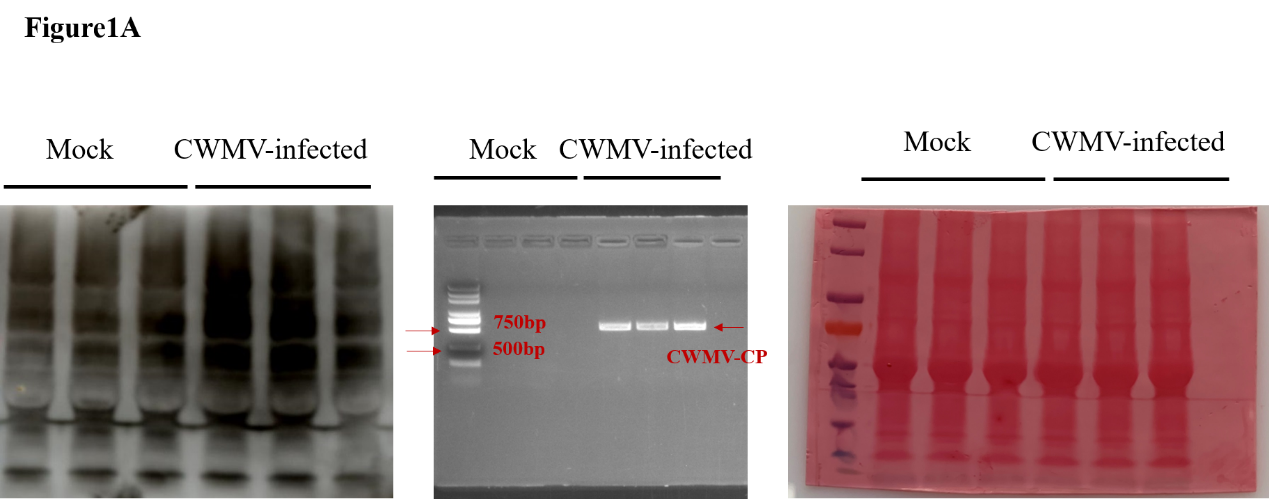


**Fig.1a Analysis of the phosphorylation level in CWMV-infected wheat.** Lanes below mock, samples were prepared form mock plants. Lanes below CWMV-infected, samples were from wheat by CWMV infection. Phosphorylation level of samples are shown in the left of figure. The detection of CWMV-CP by RT-PCR are shown in the middle of figure. Ponceau S staining loadings are shown in the right of figure.

**
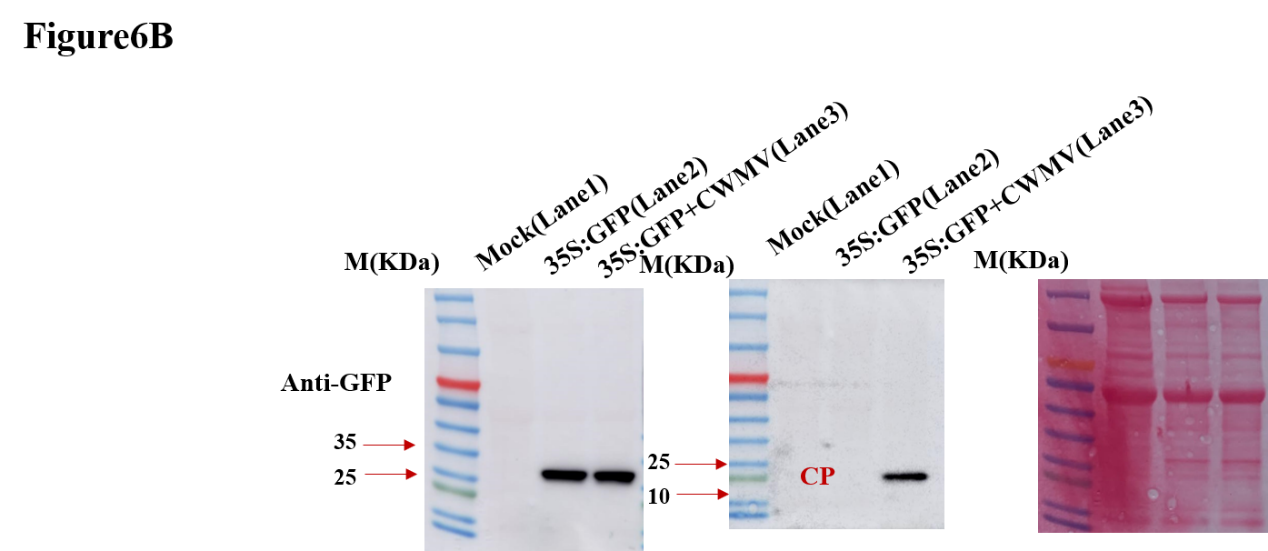
**

**Fig.6b Western-blotting assay for detecting GFP expression in *N.benthamiana*.** In vivo protein expression assay of TaChi1 and TaP5CS after CWMV infection. Lane1samples were prepared for mock (without GFP). Lane 2to 3 samples were prepared from control plants (including 35S: GFP and 35S: GFP +CWMV). The CWMV-infected samples were harvested from *N.benthamiana* by 5d post CWMV inoculation. Protein expression level of samples are shown in the left of figure. The detection of CWMV-CP by western-blotting are shown in the middle of figure. Ponceau S staining loadings are shown in the right of figure.


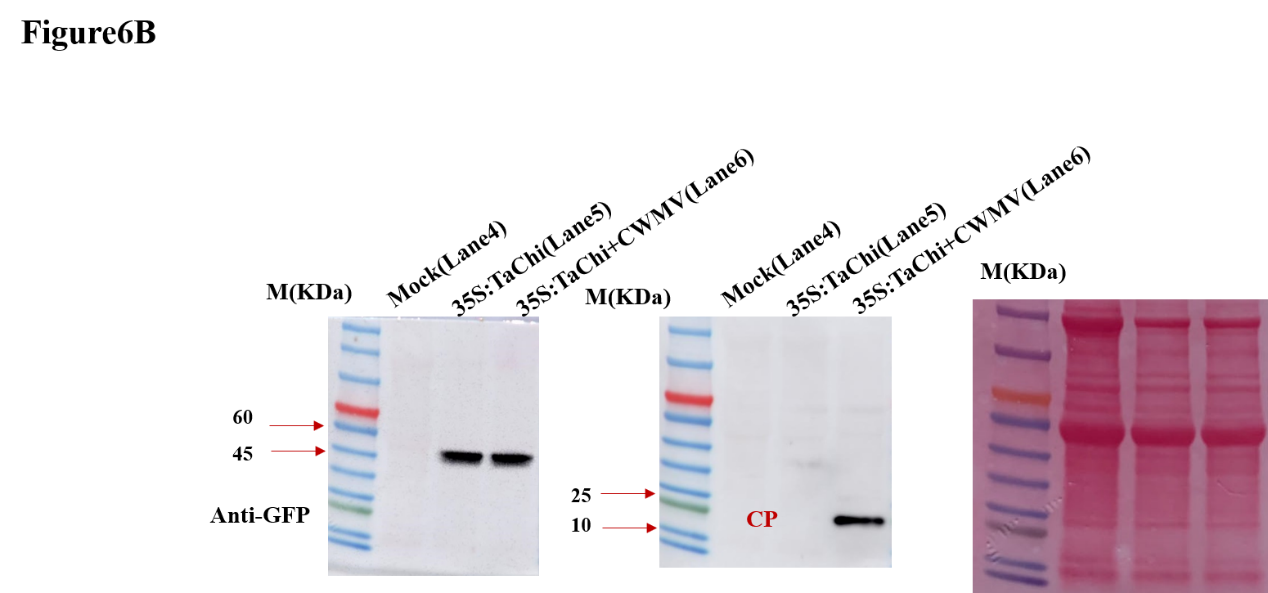


**Fig.6b Western-blotting assay for detecting TaChi1 expression in *N.benthamiana*.** In vivo protein expression assay of TaChi1 and TaP5CS after CWMV infection. Lane 4 samples were prepared for mock (without GFP). Lane 5 to 6 samples were prepared from treatment plants (including TaChi1: GFP, TaChi1: GFP+CWMV). The CWMV-infected samples were harvested from *N.benthamiana* by 5d post CWMV inoculation. Protein expression level of samples are shown in the left of figure. The detection of CWMV-CP by western-blotting are shown in the middle of figure. Ponceau S staining loadings are shown in the right of figure.


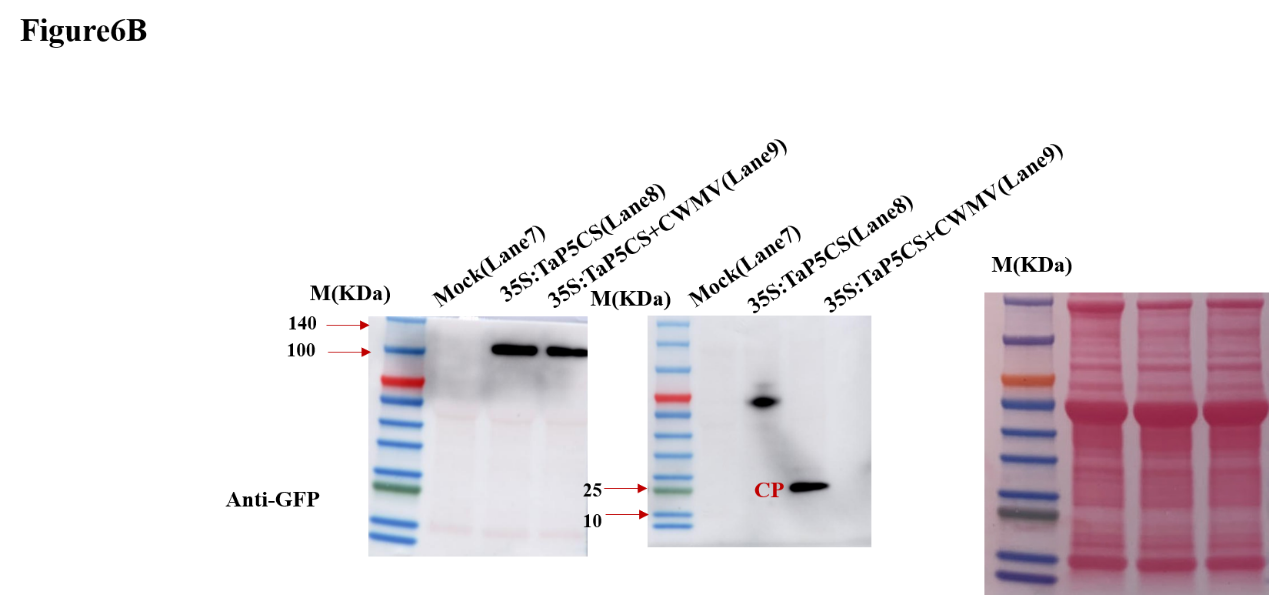


**Fig.6b Western-blotting assay for detecting TaP5CS expression in *N.benthamiana*.** In vivo protein expression assay of TaChi1 and TaP5CS after CWMV infection. Lane 7 samples were prepared for mock (without GFP). Lane 8 to 9 samples were prepared from treatment plants TaP5CS: GFP, TaP5CS: GFP+CWMV). The CWMV-infected samples were harvested from *N.benthamiana* by 5d post CWMV inoculation. Protein expression level of samples are shown in the left of figure. The detection of CWMV-CP by western-blotting are shown in the middle of figure. Ponceau S staining loadings are shown in the right of figure.


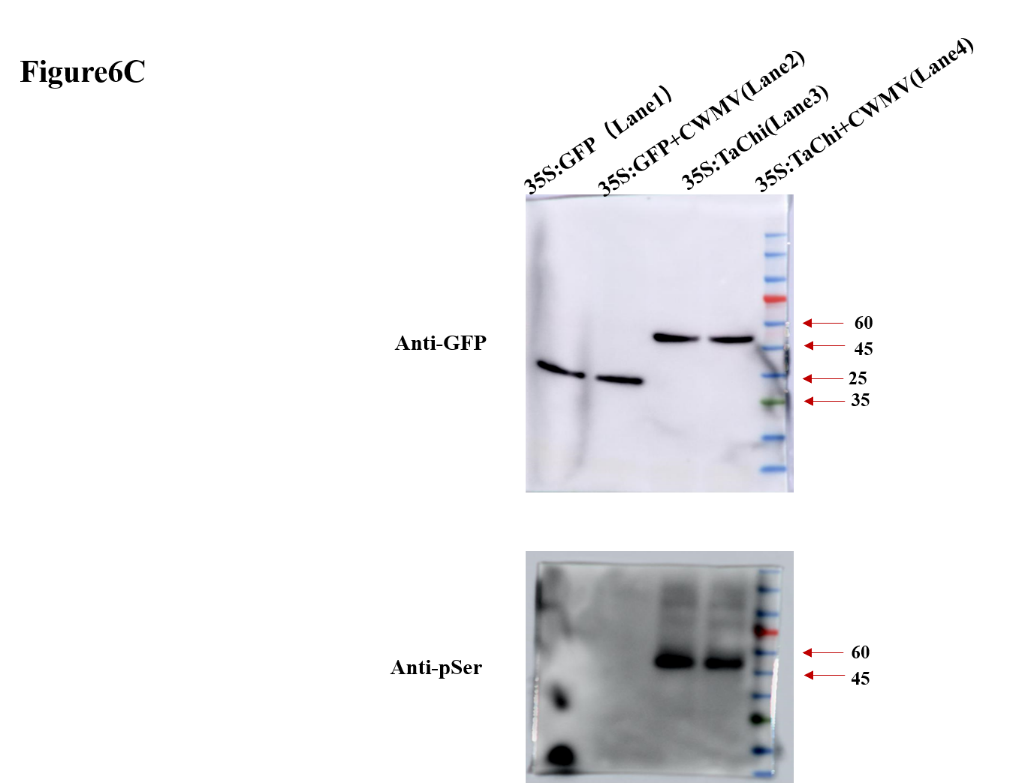


**Fig.6c Western-blotting assay for detecting TaChi1 phosphorylation in *N.benthamiana*.**

In vivo protein expression phosphorylation assay of TaChi1 after CWMV infection. Lane 1to 2 samples were prepared from control plants (including 35S: GFP and 35S: GFP +CWMV). Lane 3 to 4 samples were prepared from treatment plants (including TaChi1: GFP, TaChi1: GFP+CWMV). The CWMV-infected samples were harvested from *N.benthamiana* by 5d post CWMV inoculation. The expression level of TaChi1 is shown in the top of figure. The phosphorylation level of TaChi1 is shown in the bottom of figure.


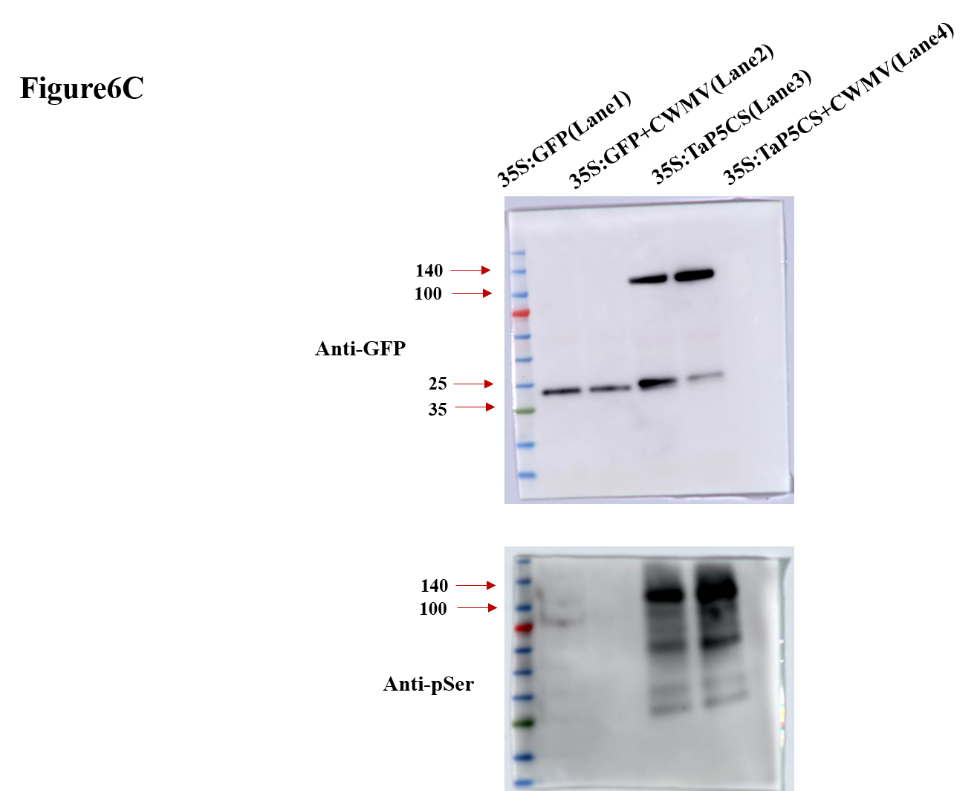


**Fig.6c Western-blotting assay for detecting TaP5CS phosphorylation in *N.benthamiana*.**

In vivo protein expression phosphorylation assay of TaP5CS after CWMV infection. Lane 1to 2 samples were prepared from control plants ((including 35S: GFP and 35S: GFP +CWMV). Lane 3 to 4 samples were prepared from treatment plants (including TaP5CS: GFP, TaP5CS: GFP+CWMV). The CWMV-infected samples were harvested from *N.benthamiana* by 5d post CWMV inoculation. The expression level of TaP5CS is shown in the top of figure. The phosphorylation level of TaP5CS is shown in the bottom of figure.


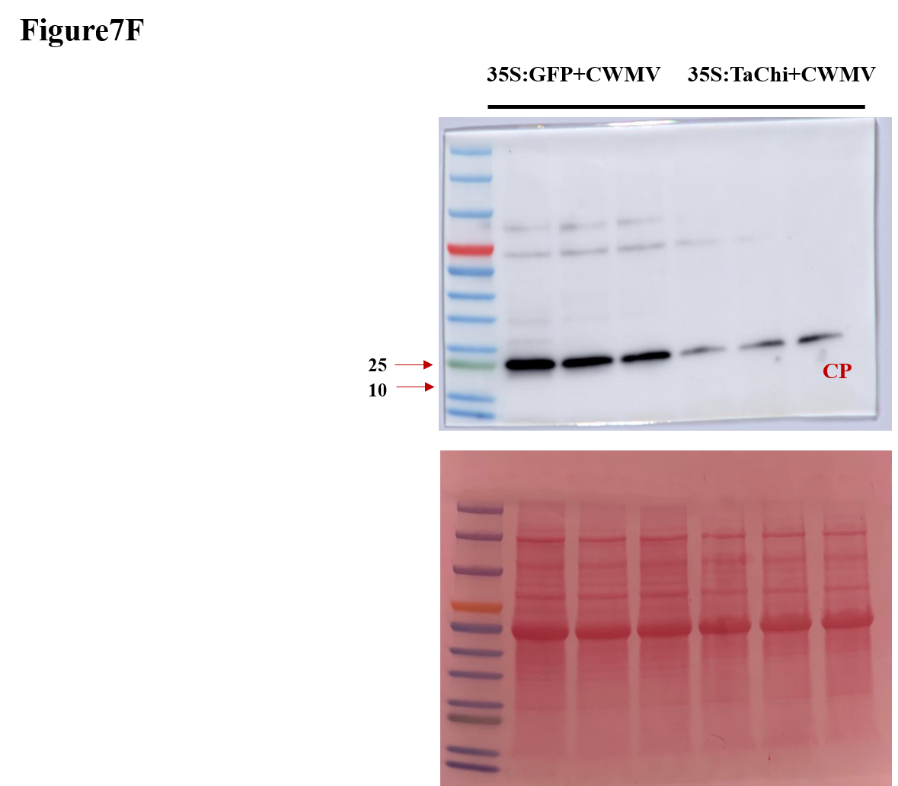


**Fig.7f Western-blotting assay for detecting CWMV-CP protein in transient overexpression TaChi1 plants.** Lanes below 35S: GFP+CWMV were control groups, and lanes below 35S: TaChi1-GFP+CWMV were treatment groups. The samples were harvested from *N.benthamiana* by 5d post CWMV inoculation. Ponceau S staining loadings are shown in the bottom of figure.


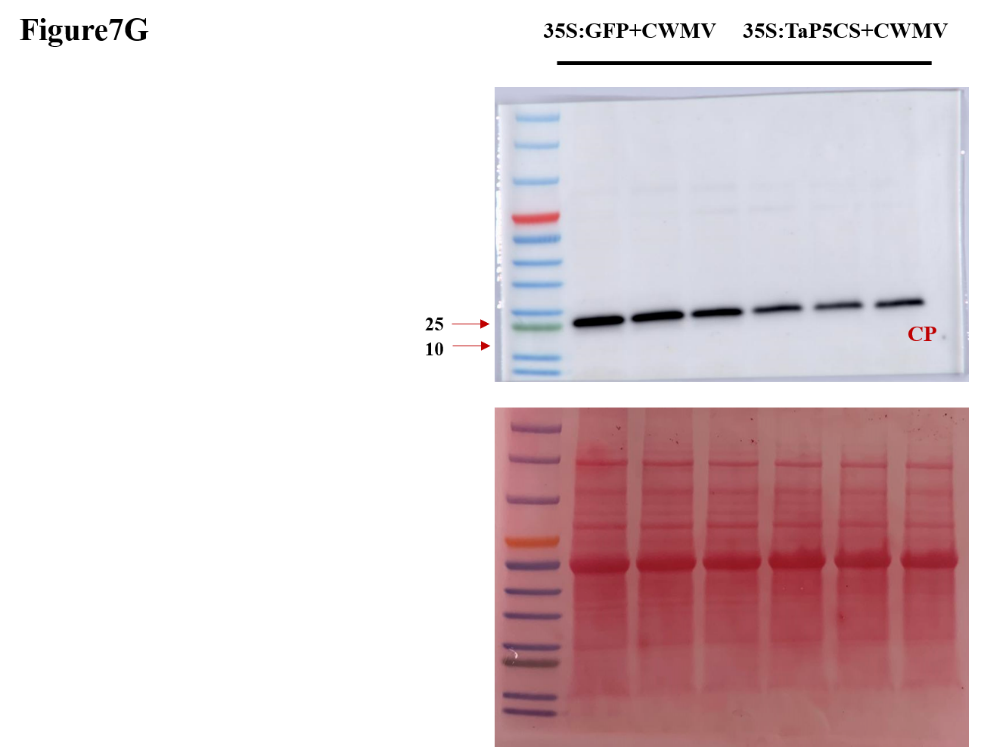


**Fig.7g Western-blotting assay for detecting CWMV-CP protein in transient overexpression TaP5CS plants.** Lanes below 35S: GFP+CWMV were control groups, and lanes below 35S: TaP5CS-GFP+CWMV were treatment groups. The samples were harvested from *N.benthamiana* by 5d post CWMV inoculation. Ponceau S staining loadings are shown in the bottom of figure.
